# Unveiling the placental bacterial microbiota: implications for maternal and infant health

**DOI:** 10.3389/fphys.2025.1544216

**Published:** 2025-03-14

**Authors:** Zhuojun Xie, Zhongsheng Chen, Yang Chai, Wang Yao, Guangyu Ma

**Affiliations:** ^1^ General Medicine Department, Clinical Medical College & Affiliated Hospital of Chengdu University, Chengdu University, Chengdu, China; ^2^ Department of Colorectal Cancer Surgery, The Second Affiliated Hospital of Harbin Medical University, Harbin, China; ^3^ Department of Obstetrics and Gynecology, The First Affiliated Hospital of Jinan University, Guangzhou, China

**Keywords:** infant health, maternal health, placental microbiota, pregnancy complications, source of placental microbiota

## Abstract

The human placenta is a unique organ that forms under specific physiological conditions and plays a crucial role in nutrient and metabolite exchange between the mother and fetus. Research on the placenta is important for understanding maternal-fetal diseases. Traditionally, the placenta was considered “sterile,” but advancements in detection techniques have revealed the presence of a low level of microorganisms. This discovery challenges the traditional notion that the uterine placenta is sterile. The revelation of this truth marks a significant breakthrough in medical research, prompting more researchers to focus on this vital organ, the placenta. Placental microbial communities may originate from the oral, vaginal, and intestinal microbiota of expectant mothers. These microorganisms may reach the maternal-fetal interface, collectively shaping the placental microbiota and contributing to the composition of normal placental microbial communities. Abnormal placental microbial communities may be associated with some pregnancy complications and fetal developmental issues such as preterm birth, gestational hypertension, fetal growth restriction, and gestational diabetes mellitus. Intervention strategies targeting microbial communities, which include modulation of placental microbiota composition or function, such as probiotics, may help prevent or treat complications related to abnormal placental microbiota during pregnancy.

## 1 Introduction

The placenta, as a crucial organ for material exchange between the mother and fetus, exists only during pregnancy. The establishment and maintenance of its integrity and functionality are essential for the survival and growth of the fetus (Sood et al., 2006). Comprising primarily the amnion, chorionic villi, and decidua basalis, capsularis, and parietalis, the placenta is formed jointly by the mother and fetus (Leviton et al., 2010). The placenta facilitates the exchange of gases, nutrients, and metabolic waste between the mother and fetus, playing a crucial role in sustaining pregnancy. It also establishes an immunological interface and influences the metabolism and development of the mother, fetus, and placenta through the production of various peptides and steroid hormones (Fichorova et al., 2011).

Traditionally, the placenta was thought to be a sterile environment; however, recent research has revealed that it is not entirely free of microorganisms. Specific microorganisms have been identified in the placental tissue, leading to a growing interest in the placental microbiota and its potential impact on fetal development and maternal-infant health (Fardini et al., 2010; Stout et al., 2013; Aagaard et al., 2014; Cao and Mysorekar, 2014; Collado et al., 2016; Prince et al., 2016; Parnell et al., 2017; Seferovic et al., 2019; Miranda-Rius et al., 2023). The presence of these microorganisms suggests that the placenta might play a role similar to other body sites, where microbial communities contribute to physiological processes. The human body’s symbiotic microbial communities play various roles in their respective ecological niches, and changes in the composition of human microbiota, such as diversity, abundance, and interrelationships among community members, have a decisive impact on the physiological functions of the entire community (Human Microbiome Project Consortium, 2012b; Human Microbiome Project Consortium, 2012a; Aagaard et al., 2013). The placental microbiota is no exception, and its composition may influence pregnancy outcomes. Abnormalities in the placental microbiota have been linked to several pregnancy-related disorders, such as preterm birth, gestational hypertension, fetal growth restriction (FGR), and gestational diabetes mellitus (GDM) (Parris et al., 2021; La et al., 2022; Saraf et al., 2022). This review will discuss recent discoveries regarding placental microbiota and their potential sources, with a particular focus on analyzing the connection between placental microbiota and pregnancy-related disorders.

## 2 Breaking tradition: discovery of the placental microbiota

Historically, it was believed that organs such as the uterus and placenta were sterile, with fetal development occurring in a germ-free environment (Aagaard et al., 2014; Braundmeier et al., 2015).

Under this paradigm, the placenta was regarded as “sterile” throughout pregnancy (Perez-Muñoz et al., 2017; Singh and Xia, 2019; Briana et al., 2021). However, recent advances in microbial detection technologies have challenged this traditional view. These microbial communities are crucial for the normal function and homeostasis of the organs and tissues they inhabit (Aagaard et al., 2014; Braundmeier et al., 2015; Garcia-Garcia et al., 2022).

Early research, such as the study by Kovalovszki et al., in 1982, found a 16% positivity rate for bacterial culture under non-inflammatory conditions in the human placenta following delivery (Kovalovszki et al., 1982). Currently, only a small fraction of symbiotic bacteria can be successfully cultured *ex vivo*, while the majority remain unculturable. Consequently, conventional bacterial culture may underestimate placental microbial detection. Past studies primarily employed morphological methods to preliminarily observe bacterial presence in different placental regions (Goldenberg et al., 2000; Romero et al., 2008; Fardini et al., 2010; Stout et al., 2013). One study involving 195 patients found various Gram-positive and Gram-negative intracellular bacteria in 27% of placental basal plates (Stout et al., 2013). Cao et al. identified diverse bacteria morphologies within trophoblastic cells (Cao and Mysorekar, 2014), further confirming bacterial presence in the placenta, given its development from trophoblastic cells. However, morphological methods have limitations in studying placental microbiota. Many microbes remain unculturable under standard aerobic laboratory conditions (Perez-Muñoz et al., 2017; Heil et al., 2019), which may result in traditional methods underestimating bacterial presence in low-biomass samples like healthy placentas.

The advent of high-throughput sequencing greatly enhances microbial detection sensitivity, accuracy, and comprehensive assessment of placental microbial diversity and species abundance (Aminu et al., 2023). High-throughput sequencing technologies, such as 16S rRNA gene sequencing and metagenomic sequencing, allow for a more detailed and comprehensive analysis of the placental microbiota compared to traditional methods. 16S rRNA gene sequencing, which targets a specific region of the bacterial rRNA gene, enables the identification of bacterial species in the placental microbiota. Metagenomic sequencing, which sequences the entire genetic material, provides a more complete view by capturing not only bacterial species but also fungi, viruses, and other microorganisms. These sequencing methods have overcome many of the limitations associated with traditional bacterial culture and morphological methods, allowing for the detection of microbial DNA in the placenta (Collado et al., 2016; Prince et al., 2016; Seferovic et al., 2019) amniotic fluid, and even umbilical cord blood (Moore et al., 2017; Kyono et al., 2018). This has led to the development of the “*in utero* colonization” theory, which suggests that the uterine and placental microbiota contribute to the colonization of fetal organs before birth. Studies using high-throughput sequencing have detected microbiota in umbilical cord blood, amniotic fluid, placenta, and fetal membranes (Jiménez et al., 2005; Rautava et al., 2012b). These studies have revealed that the placenta is not “sterile” but contains specific microbial communities, primarily consisting of bacterial phyla such as *Firmicutes*, *Tenericutes*, *Proteobacteria*, *Bacteroidetes*, and *Fusobacteria*. Although these microbes are present in low abundance, their species composition differs significantly from that of other human body sites (Aagaard et al., 2014; Zheng et al., 2015; Bassols et al., 2016). The microbial diversity in the placenta detected by these high-throughput methods is much greater than what could be identified using traditional techniques, with a wider range of bacterial taxa being detected in comparison to previous studies based on culture or morphological analysis.

The aforementioned studies confirm that the placenta is not “sterile” but harbors specific microbial communities. This finding prompts further inquiry: What is the source of placental microbiota? Are these microbes intrinsic to the placenta? If so, when do they colonize the placenta? Do the microbial inhabitants of the placenta possess functionality? Present research has yet to fully elucidate the functional roles of placental microbiota, indicating a need for further investigation to understand their impact on pregnancy and fetal development.

## 3 Origin of placental microbiota

**FIGURE 1:**
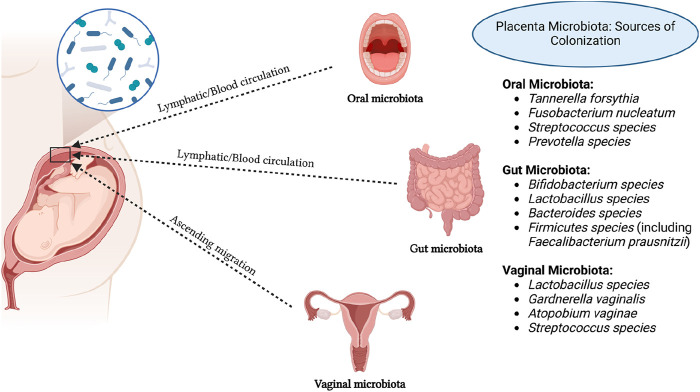
Potential sources of placental microbiota colonization. Created in https://BioRender.com.

The origins of placental microbiota colonization are the subject of ongoing discussion, with several proposed sources including ascending migration from the vagina (Goldenberg et al., 2008), hematogenous dissemination from the gut (Perez et al., 2007; Satokari et al., 2009), and oral cavity (Fardini et al., 2010) (Figure 1). Research suggests that approximately half of the placental microbiota can be detected in samples from the mother’s vagina, rectum, and oral cavity, which hints at possible routes of microbial transfer (Liu et al., 2019). Table 1 summarizes the evidence supporting these potential sources of placental microbiota. Regarding sample collection procedures, microbiota samples were typically collected from the placenta using sterile techniques. Placental tissues were either swabbed or biopsied to extract microbial DNA for sequencing. Samples from the gut, vaginal, and oral microbiota were collected using standard protocols: fecal samples were obtained with sterile collection kits, vaginal swabs were taken using sterile cotton swabs, and oral microbiota was collected using saliva samples or buccal swabs. For the literature search, we used the following search terms: “placental microbiota”, “microbial colonization”, “ascending migration”, “vaginal microbiota”, “gut microbiota”, “oral microbiota”, “hematogenous dissemination”, “microbial transfer”, “microbiome transfer routes”, “placental microbial DNA”, and “oral-gut-placenta axis”. The search string included combinations such as (“placental microbiota” AND “microbial colonization”) OR (“ascending migration” OR “hematogenous dissemination” OR “oral microbiota”) AND (“microbial transfer” OR “placental microbial DNA”) AND (“vaginal microbiota” OR “gut microbiota” OR “oral microbiota”). The search was conducted using several databases, including PubMed and Web of Science.

**TABLE 1:** The evidence that placental microbiota origin.

References	Year	Study population (Gestational week)	Methods of microorganism identification	Source of placental microbiota	Experimental protocol	Research findings
Aagaard et al. (2014)	2014	320 (Preterm Cases: 34.9 ± 2.9 weeks, Term Cohort: 39.4 ± 1.1 weeks)	16S rDNA and WGS	Oral microbiota	Collected placental specimens under sterile conditions and compared them with oral, skin, nasal, vaginal, and intestinal microbiota of non-pregnant controls	The placenta predominantly contains non-pathogenic *Escherichia coli*, and oral bacteria like *Tannerella forsythia* and *Fusobacterium nucleatum*. Placental microbiota closely resembles oral microbiota
Hu et al. (2021)	2021	40 (Normal: 39.5 weeks, IUGR: 36.7 weeks)	16S rRNA	Oral microbiota	Used SourceTracker to assess contributions from oral, vaginal, and gut microbiota. Compared with weighted and unweighted UniFrac distances	Oral microbiota contributes most to placental microbiota, showing greater similarity to oral microbiota than gut or vaginal microbiota
Gomez-Arango et al. (2017)	2017	37 (Mean gestational age: 39.4 (38.6–40.3) weeks)	16S rRNA	Oral and gut microbiota	Collecting microbiota samples from the intestine, oral cavity, and placenta of the same pregnant woman to assess the contribution of fecal and oral microbiota to the establishment of placental microbiota	The placental microbiota demonstrates a higher similarity to the oral microbiota, suggesting that both oral and intestinal microbiota contribute to the seeding of the placental microbiota. Thus, oral and intestinal microbial communities may serve as sources for the placental microbiota
Han et al. (2006)	2006	34 (−)	16S and 23S rRNA	Oral microbiota	PCR was utilized to examine subgingival plaque, vaginal swabs, and amniotic fluids collected from 34 pregnant women, employing universally conserved primers targeting 16S and 23S rRNA.	A single colony of *Bergeyella* isolated showed 100% similarity at the level of 16S and 23S rRNA sequences with samples obtained from subgingival plaque and amniotic fluid sources from patients
Han et al. (2004)	2004	-	-	Oral microbiota	Injecting *Fusobacterium nucleatum* intravenously into pregnant mice	High levels of *Fusobacterium nucleatum* were detected in the placenta of mice, leading to preterm birth and term stillbirths in pregnant mice
Makino et al. (2011)	2011	8 pairs of mothers and infants (−)	Multilocus sequencing typing (MLST) and amplified fragment length polymorphism (AFLP) analysis	Gut microbiota	Two hundred seven isolates from 8 pairs of mothers and infants were discriminated by multilocus sequencing typing and amplified fragment length polymorphism analysis	There were 11 strains of highly homologous *Bifidobacterium* species detected in the feces of both pregnant mothers and infants, suggesting that *Bifidobacterium* may transfer from the maternal gut to the placenta through a certain pathway
Satokari et al. (2009)	2009	34 (−)	Species-specific PCR for the detection of DNA.	Gut microbiota	Investigated human placental samples (25 vaginal deliveries, 9 cesarean sections) for *Lactobacillus* and *Bifidobacterium* presence	*Bifidobacterium* DNA was detected in 33 out of 34 placental samples, while *Lactobacillus* DNA was detected in 31 out of 34 placental samples
Mitchell et al. (2020)	2020	-	-	Gut microbiota	Orally inoculated pregnant mice with genetically labelled *E. fecium* from breast milk	Genetically labelled *E. fecium* detected in initial meconium of pup’s post-cesarean delivery
Li et al. (2024)	2024	31 pregnant individuals and their offspring (Full-term pregnancy-)	16S rRNA	Gut and vaginal microbiota	Collected placental specimens at term; compared with vaginal and intestinal microbiota at 32–34 weeks and at term	Placental microbiota shows specific dominance. Correlated with gut and vaginal microbiota at 32–34 weeks but not at full term

### 3.1 Oral microbiota of pregnant women

Intrauterine infection occurs when pathogenic microorganisms ascend from the vagina into the “sterile” uterus. However, increasing evidence suggests that many pathogens responsible for intrauterine infection are not limited to vaginal microbiota. For instance, microorganisms such as *F. nucleatum* and species from *Bergeyella*, *Eikenella*, and *Capnocytophaga spp*. are found in the oral cavity (Hill, 1998; Han et al., 2006; 2009). Recent macrogenomic studies on placental microbiota have shown similarities between placental microbial characteristics and oral microbiota. The most abundant microorganisms in the placenta are non-pathogenic *Escherichia coli* commonly found in the intestine and two oral bacteria (*Tannerella forsythia* and *Neisseria*). This suggests that placental microbiota may originate from the oral cavity (Aagaard et al., 2014). Nevertheless, these studies typically compare placental microbiota with the symbiotic microbiota of non-pregnant individuals rather than comparing the microbiota of the same pregnant woman during pregnancy. Studies that collect microbiota from the intestines, oral cavity, and placenta of the same pregnant women have evaluated the contribution of fecal and oral microbiota to placental microbiota. These studies found a higher similarity between placental microbiota and oral microbiota (Gomez-Arango et al., 2017). However, a limitation of these studies is the exclusion of vaginal microbiota from the analysis.

The correlation between oral and placental microbiota suggests a significant connection, despite their distinct ecological niches. SourceTracker is a Bayesian method used to estimate the contribution of different source environments to a target microbial community by comparing their similarity. It is commonly applied in microbiome studies to identify the origins of microbiota in specific locations, such as the placenta. It is commonly used in microbiome studies to assess the contribution of microbiota from various body sites to a particular microbial environment, such as the placenta. FEAST (Fast Expectation-Maximization for microbial community profiling) is another tool that uses an Expectation-Maximization algorithm to predict microbial sources. FEAST is more efficient in handling complex, high-dimensional data and provides more accurate source predictions by utilizing additional information and constraints, making it potentially superior to SourceTracker in certain cases (Knights et al., 2011). In one study utilizing SourceTracker, contributions of the microbiota from the oral cavity, vagina, and intestines to the placental microbiota were evaluated. Weighted and unweighted UniFrac distances were used to compare differences in microbiota composition among the oral cavity, vagina, intestines, and placenta. The study found that the oral microbiota made the largest contribution to the placental microbiota. Placental microbiota were more similar to oral microbiota than to intestinal or vaginal microbiota (Hu et al., 2021). In another study, Han et al. detected a strain of *Bergeyella* in a group of clinical samples resembling chorioamnionitis (Han et al., 2006). This strain showed 100% similarity at the 16S and 23S rRNA sequence levels to samples from patients’ subgingival plaques and amniotic fluid sources. However, this strain was not detected in vaginal samples. Notably, *Bergeyella* has been reported to exist only in the human oral cavity. Furthermore, Redline et al. induced preterm birth and stillbirth in mice by intravenous injection of the Gram-negative anaerobic oral bacterium *Fusobacterium nucleatum* (Han et al., 2004). Significantly, a large amount of *Fusobacterium nucleatum* was detected in the mouse placenta, suggesting that preterm birth and stillbirth may be due to local infection by this bacterium.

The specific pathways by which oral microbiota enter the placenta remain to be fully understood. Given that some oral microbiota can bind to vascular endothelium and alter its permeability (Han et al., 2000; Fardini et al., 2011), it is plausible that they may enter the placenta via hematogenous dissemination. Oral microbiota could first enter the bloodstream of pregnant women and then migrate to the placenta, similar to the dissemination of *E. coli* to other organs via the bloodstream. However, the exact timing of this colonization—whether during early vascularization and embryo implantation or after placental formation-and the related mechanisms require further investigation.

### 3.2 Gut microbiota of pregnant women

One study assessing the contributions of fecal and oral microbiota to the establishment of placental microbiota found that both oral and intestinal microbiota could be sources of placental microbiota, but oral microbiota were the main contributors (Gomez-Arango et al., 2017). Another study showed that placental microbiota were associated with the intestinal and vaginal microbiota of women at 32–34 weeks of gestation but not with those at full term (Li et al., 2024). This finding provides evidence supporting the possibility of maternal intestinal and vaginal microbiota transmitting to the placenta *in utero*. In a study, pregnant mice were orally administered genetically labeled *E. faecium*, and genetically labeled *E. faecium* was detected in the initial feces of offspring after cesarean section (Jiménez et al., 2008). *Bifidobacteria* and *Lactobacilli* are part of the normal human intestinal microbiota and may transfer to the placenta. In another study, the presence of *Bifidobacterium* and *Lactobacillus* DNA was detected in 34 human placenta samples (25 vaginal deliveries and 9 cesarean sections), suggesting horizontal transfer of bacterial DNA from mother to fetus through the placenta (Satokari et al., 2009). Makino et al. found 11 highly homologous strains of *Bifidobacterium* in maternal and infant feces (Makino et al., 2011), suggesting that *Bifidobacteria* may transfer from the maternal intestine to the placenta and then to the infant intestine, proposing the hypothesis of “gut-to-extraintestinal colonization” of maternal-infant microbiota transmission (Jiménez et al., 2008). Dendritic cells may play an important role in this process (Perez et al., 2007). Dendritic cells can penetrate the intestinal epithelial mucosa, directly absorb bacteria from the intestinal lumen, enter the maternal lymph/blood circulation through the mucosa-associated lymphoid tissue of the immune system, and then enter the maternal-fetal interface, jointly constructing placental microbiota (Perez-Muñoz et al., 2017). In an animal experiment involving cows, microbial communities in blood, feces, and uterine samples were analyzed using 16S rRNA sequencing technology, revealing similar pathogens in blood and uterus, supporting the view that intestinal microbiota may spread to the uterus via the bloodstream (Jeon et al., 2017). Based on these findings, it can be inferred that placental microbiota may originate from the oral cavity, vagina, and intestine of pregnant women. During pregnancy, the permeability of the intestinal and oral epithelium increases, allowing microbiota from multiple reservoirs, such as the oral cavity and intestine, to enter the maternal lymph/blood circulation and colonize placental tissues, becoming important components of normal placental microbiota.

### 3.3 Vaginal microbiota of pregnant women

The vaginal microbiota of pregnant women hosts a variety of microorganisms and maintain a dynamic balance with the microenvironment, playing a crucial role in maintaining maternal-infant health. Given the proximity of the vaginal microbiota to the uterus, the vagina serves as a rich reservoir of microbiota that can ascend to higher locations (Goldenberg et al., 2008). Studies have indicated a strong correlation between placental microbiota and vaginal microbiota (Li et al., 2024). Ascension of microbiota from the birth canal is one of the significant ways in which the placenta acquires its microbial community (Goldenberg et al., 2008). Researchers have observed changes in the vaginal, blood, and placental microbiota during pregnancy, noting a trend of increased ascension of birth canal microbiota into the bloodstream, which then circulates to placental tissues. Additionally, lactobacilli levels were found to be positively correlated with gestational age (MacIntyre et al., 2015). Studies have detected *Mycoplasma* and *Gardnerella* in chorionic membranes and *Mycoplasma* and *Bergeyella* in amniotic fluid, indicating the possibility of colonization in the placenta by these microorganisms commonly found in the vagina, often associated with chorioamnionitis and preterm birth (DiGiulio et al., 2010). In comparison to oral microbiota, research on the correlation between vaginal microbiota of pregnant women and placental microbiota is relatively limited, and the hypothesis of placental microbiota originating from the vaginal microbiota of pregnant women requires further confirmation with substantial data. It remains unknown whether vaginal microbiota are original or symbiotic colonizers of the placenta and the mechanism by which they migrate to the placenta, whether via ascending infection or penetration through epithelium followed by migration through the bloodstream system, warrants further investigation.

## 4 Placental microbiota and maternal-fetal immune interface

The placental microbiota plays a crucial role in maintaining immune tolerance balance during pregnancy, as well as in the occurrence of allergic diseases and the growth and development of the nervous system in offspring (Mor and Kwon, 2015). The maintenance of a successful pregnancy is inseparable from the immune system balance at the maternal-fetal interface, where the placenta acts as a tightly regulated immune organ containing a large number of immune cells, such as natural killer cells, macrophages, T cells, and decidual stromal cells with immunomodulatory functions (Erlebacher, 2013). These immune cells are essential for maintaining normal pregnancy and maternal-fetal immune tolerance.

Certain bacteria in the gut microbiota, such as *Lactobacilli*, *Bifidobacteria*, and *Escherichia coli*, can stimulate antigen-presenting cells to secrete immunologically active cytokines such as IL-10, IL-12, IL-16, and TNF, which downregulate hypersensitivity reactions, regulate the balance between Th1 and Th2 responses, and induce immune tolerance (Karlsson et al., 2004). Segmented filamentous bacteria can induce Th-17 in the intestines, regulating the host’s immune system (Ivanov et al., 2009). Symbiotic bacteria in the gut epithelium can inhibit the NF-kB pathway and the connection between NOD2 and peptidoglycans stimulated by Toll-like receptors, leading to the downregulation of pro-inflammatory cytokines (Mortha et al., 2014; Ridaura and Belkaid, 2015).

The gut microbiota plays a crucial role in stimulating and regulating both local intestinal immunity and systemic immune responses. It is speculated that the placental microbiota may mediate important factors in stimulating and feedback regulation of placental local immunity and maternal systemic immunity. However, there is limited understanding of the relationship between placental microbiota during pregnancy and immune regulation. Only some related studies have shown that nourishing cells can recruit and activate macrophages with antigen-presenting capabilities, prompting them to secrete cytokines and chemokines. Additionally, nourishing cells can also influence macrophage responses to lipopolysaccharides (Fest et al., 2007). The placental microbiota stimulates local immunity in the placenta, primarily by balancing the ratio of CD56^+^CD16+/CD56+CD16 lymphocytes and placental macrophages, thus providing immune protection and nutritional support. Further research is needed to delve into the mechanisms of interaction between placental microbiota and the placental local immune system (Yang P. et al., 2024). This includes studying how microorganisms influence the differentiation, activity, and function of immune cells, as well as how immune cells regulate the growth and metabolism of microorganisms.

Placental microbiota dysbiosis has activated the maternal immune system, inducing the release of inflammatory cytokines such as TNF-α and IL-6, which have in turn upregulated the expression of XO. As a key enzyme in purine metabolism, XO has catalyzed the conversion of hypoxanthine and xanthine into uric acid while simultaneously producing reactive oxygen species (ROS), directly exacerbating oxidative stress (Németh et al., 2002; Annesi et al., 2024). This dual role has made XO a central molecule linking inflammation and oxidative stress. Clinical studies have suggested that abnormal XO activity has been closely associated with various obstetric diseases: in patients with gestational hypertension, elevated plasma XO activity has correlated positively with levels of uric acid and lipid peroxidation products, indicating its potential as an early marker of endothelial dysfunction (Németh et al., 2002); in preeclampsia, excessive XO activation induced by placental ischemia-reperfusion has mediated endothelial injury through ROS (Doehner and Landmesser, 2011; Annesi et al., 2024); in GDM, oxidative stress mediated by XO has disrupted the insulin signaling pathway, while the hyperglycemic environment has further stimulated XO activity, creating a vicious metabolic cycle (Németh et al., 2002). As a dual biomarker of inflammation and oxidative stress, xanthine oxidase has provided a new perspective for the early diagnosis and intervention of obstetric diseases associated with placental microbiota dysbiosis. Future research should further validate its clinical translational potential and explore multidimensional therapeutic strategies based on the XO pathway.

## 5 Placental microbiota and pregnancy-related disorders: exploring and understanding from a microbial community perspective

**FIGURE 2:**
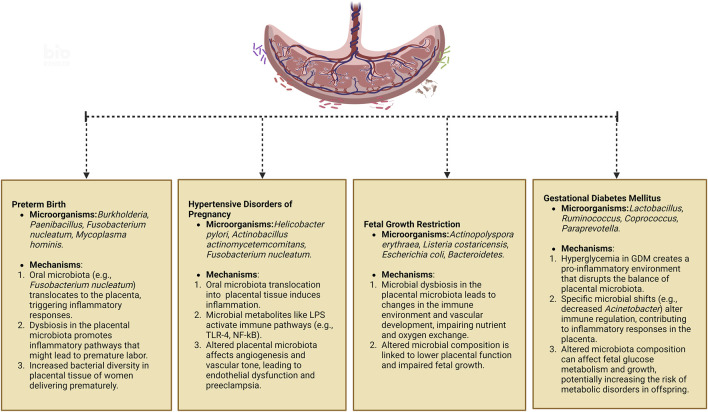
Pathophysiological pathways linking maternal microbiota to pregnancy complications and neonatal outcomes. Created in https://BioRender.com.

The placental microbiota, a vital microbial ecosystem during pregnancy, has garnered significant attention for its potential role in pregnancy-related diseases. Imbalances within this microbiota may contribute to a range of pathophysiological abnormalities, thereby increasing the risk of complications during pregnancy (Stupak and Kwaśniewski, 2023; Yang P. et al., 2024). Emerging research has shown that disruptions in the placental microbiota may be closely linked to the onset and progression of conditions such as preterm birth, gestational hypertension, FGR, GDM, and other related complications (Barak et al., 2007; Mor and Kwon, 2015; Zheng et al., 2015; 2017; Bassols et al., 2016; Pelzer et al., 2017; Seferovic et al., 2019; La et al., 2022). Table 2 summarizes the specific changes in the placental microbiota that have been associated with these disorders. Figure 2 shows the pathophysiological pathways linking maternal microbiota to pregnancy complications and neonatal outcomes.

**TABLE 2:** The placental microbiota changes associated with pregnancy-related disorders.

References	Year	Sample size	Delivery mode	Species	Pregnancy-related disorders	Changes in placental microbiota
Aagaard et al. (2014)	2014	Preterm Cases = 89 Term Cohort = 231	Vaginal and cesarean delivery	Homo	Preterm Birth	In preterm placenta samples, *Burkholderia* is more abundant, while *Paenibacillus* is more abundant in full-term placenta. Increased relative abundance of *Actinomycetales* and *Alphaproteobacteria* in preterm placenta
Doyle et al. (2014)	2014	Preterm Cases = 14 Term Cohort = 10	Vaginal and cesarean delivery	Homo	Preterm Birth	*Mycoplasma hominis*, *Aerococcus christensenii*, *Gardnerella vaginalis*, and *Fusobacterium nucleatum* are either exclusively present or significantly more abundant in preterm compared to full-term gestation
Amarasekara et al. (2015)	2015	Preeclampsia Cases = 55 Control Cases = 55	Cesarean delivery	Homo	Preeclampsia	Placental organisms include *Bacillus cereus*, *Listeria*, *Salmonella*, *Escherichia*, *Klebsiella pneumoniae*, *Anoxybacillus*, *Variovorax*, *Prevotella*, *Porphyromonas*, and *Dialister*
Stupak and Kwaśniewski (2023)	2023	FGR Cases = 18 Control Cases = 18	Cesarean delivery	Homo	FGR	In the FGR group, increased levels of *Actinopolyspora erythraea*, *Listeria costaricensis*, *E. coli*, *Methylobacterium*, *Acidobacteria bacterium*, *Bacteroidetes bacterium*, *Paenisporsarcina* sp., *Thiodiazotropha endol oripes*, and *Clostridiales bacterium* were detected. In the normal group, *Flavobacterial bacterium*, *Aureimonas* sp., and *Bacillus cereus* showed increased abundance
Hu et al. (2021)	2021	IUGR Cases = 20 Control Cases = 20	Vaginal and cesarean delivery	Homo	IUGR	Alpha and beta diversity were compared, and differential taxa features associated with IUGR were identified. Microbial screening of the placenta demonstrated a diverse range of flora predominantly including *Proteobacteria*, *Fusobacteria*, *Firmicutes*, and *Bacteroidetes*. Neither alpha- nor beta-diversity was significantly different by IUGR status. However, at the taxa level, IUGR patients had a significantly higher prevalence of *Neisseriacea*
Zheng et al. (2015)	2015	Low Birth Weight (LBW) Cases = 12 Normal Birth Weight (NBW) Cases = 12	Vaginal delivery	Homo	LBW	At the phylum level, *Fusobacteria* was significantly increased and *Cyanobacteria* was significantly decreased in LBW group, compared with NBW group. It is indicated that the relative abundance of *Lactobacillus*, *Clostridium_sensu_stricto_1*, *Cyanobacteria_unclassified*, *Ruminococcus*, *Lawsonia* and *Cyanobacteria_norank* were significantly lower in LBW group at the genus level. Conversely, the relative abundance of *Megasphaera*, *Faecalibacterium*, *DSSF69_norank*, *Jeotgalicoccus*, *Pediococcus*, *Sneathia*, and *Sphingobacterium* were significantly higher in LBW group, compared with NBW group
Bassols et al. (2016)	2016	GDM Cases = 11 Contral Cases = 11	Vaginal and cesarean delivery	Homo	GDM	In women with GDM, there is a decrease in the abundance of the order *Pseudomonadales*and the genus *Acinetobacter*in the placenta
Tang et al. (2020)	2020	GDM Cases = 8 Control Cases = 7	Vaginal and cesarean delivery	Homo	GDM	The placental microbiota, in both GDM and control groups, primarily consists of four phyla: *Bacteroidetes*, *Firmicutes*, *Actinobacteria*, and *Proteobacteria*. In the placentas of women with GDM compared to controls, there are specific differences in genus abundance, with higher levels of *Ruminococcus*, *Coprococcus*, *Paraprevotella*, and *Lactobacillus*, and lower levels of *Veillonella*
Zheng et al. (2017)	2017	GDM Cases = 10 Control Cases = 10	Cesarean delivery	Homo	GDM	In both the GDM and non-GDM groups, the primary phyla were *Proteobacteria*, *Bacteroidetes*, *Actinobacteria*, and *Firmicutes*, with *Proteobacteria* being the most abundant. However, in the GDM group, there was an increase in the proportion of *Proteobacteria* and a decrease in the proportions of *Bacteroidetes*, *Actinobacteria*, and *Firmicutes* compared to the controls

### 5.1 Placental microbiota and preterm birth

Premature birth, defined as delivery occurring between 28 and less than 37 weeks of gestation, is the leading cause of morbidity and mortality among newborns (Prince et al., 2014a). Babies born during this period are termed premature infants. The causes of preterm birth are multifactorial and can include infections, maternal hypertension, placental complications, and other factors. Stout et al. discovered bacteria on the basal plate of placentas from premature infants. The basal plate refers to the peripheral area of the placenta near the maternal side, composed of tissue layers below the interface between the mother and fetus, and the types of bacteria detected were also found in the extravillous trophoblast cells positive for human leukocyte antigen-G (HLA-G) (Red-Horse et al., 2004; Stout et al., 2013). Studies have found that the placental microbiota in preterm pregnancies differs from that in normal pregnancies, suggesting an association between placental microbiota and preterm birth. In pregnant women delivering prematurely, *Burkholderia* showed higher abundance, whereas *Paenibacillus* was more abundant in full-term placenta samples. Advanced taxonomic analysis using phylogenetic trees revealed increased relative abundance of *Actinomycetales* and *Alphaproteobacteria* in preterm placenta (Aagaard et al., 2014). Microorganisms such as *Mycoplasma hominis*, *Aerococcus christensenii*, *Gardnerella vaginalis*, and *F. nucleatum* were either present only in preterm fetal membranes or had significantly higher abundance during preterm birth compared to full-term pregnancies (Doyle et al., 2014).

In the placental tissue of women undergoing premature delivery, there is a broader and more diverse range of bacterial species present, which may exist without obvious complications. However, it is currently unclear whether bacterial load and diversity have any impact on triggering preterm birth (Doyle et al., 2014). The oral microbiota may be one of the causes leading to changes in placental microbiota and triggering preterm birth. Han et al. detected *Bergeyella* in the amniotic fluid of women experiencing preterm birth, and analysis of 16S rDNA sequences revealed consistency with subgingival plaque in the women’s mouths, while it was not detected in the vagina, suggesting a potential association between *Bergeyella* and preterm birth (Han et al., 2006). Redline et al. induced premature birth and stillbirth in mice by intravenously injecting them with the Gram-negative anaerobic bacterium *Fusobacterium nucleatum* from the oral cavity, suggesting that these bacteria may reach the placenta through the bloodstream or other pathways, triggering an inflammatory response and thus preterm birth (Han et al., 2004). Epidemiological studies have also shown that pregnant women with periodontal disease are more likely to experience preterm birth, with pathogenic bacteria associated with periodontal disease found in the amniotic fluid of these women (Prince et al., 2014b). This further supports a possible connection between oral microbiota and preterm birth. In conclusion, the placental microbiota appears to play a significant role in preterm birth. Although the precise relationship between placental microbiota and preterm birth is not yet fully understood, these findings suggest that microorganisms may contribute to preterm birth, offering important directions for future research. Further investigation is needed to elucidate how microorganisms impact the placenta and pregnancy, potentially leading to preterm birth. This may involve multiple mechanisms, including microbial metabolites, immune responses, inflammation, and other related factors.

### 5.2 Placental microbiota and hypertensive disorders of pregnancy

Preeclampsia is a common and serious complication during pregnancy, typically occurring after 20 weeks of gestation and characterized by symptoms such as high blood pressure, proteinuria, and edema (Rubio Gonzalez et al., 2024; Thadhani et al., 2024). Currently, abnormal remodeling of the spiral arteries in the placenta, oxidative stress at the maternal-fetal interface, and systemic inflammatory responses are considered to be the main pathological features leading to endothelial cell damage in preeclampsia (Kornacki et al., 2023; Yang M. et al., 2024). Recent studies have shown that gut microbiota is related to hypertensive disorders in pregnancy (Wu et al., 2023). Gut microbiota is essential for blood pressure (BP) homeostasis and vascular function (Witkowski et al., 2020). Germ-free rats exhibit hypotension and reduced vascular contractility, showing that microbiota play a critical role in BP regulation (Joe et al., 2020). Reintroducing microbiota restored both BP and vascular contractility, highlighting microbiota’s vascular influence. Further, actin polymerization in arterial smooth muscle was stabilized in conventionalized GF rats, linked to increased cofilin phosphorylation, suggesting that microbiota regulate vascular tone via actin dynamics. These results reveal gut microbiota as a key regulator of vascular function and BP. TMAO, produced by gut microbiota, is linked to hypertension. In mice, TMAO enhanced Ang II-induced hypertension by increasing blood pressure and vasoconstriction. This effect was reduced by antibiotics, indicating a microbiota-dependent mechanism (Jiang et al., 2021). TMAO increased calcium release in vascular smooth muscle cells, activating the PERK/ROS/CaMKII/PLCβ3 pathway. Inhibition of this pathway blocked TMAO’s effects. Studies have shown a direct correlation between arterial stiffness and the abundance of bacteria linked to increased gut permeability and inflammation (Agnoletti et al., 2022). In contrast, arterial stiffness is inversely related to microbiota diversity and the presence of bacteria associated with a healthier microbiota composition.

Recent studies suggest that alterations in the placental microbiota may also play a role in the development of preeclampsia (Olaniyi et al., 2020; Ishimwe, 2021). Researchers are investigating the microbial communities in preeclamptic placentas to identify specific pathogenic and commensal microorganisms and understand their interactions. For example, pro-inflammatory bacteria such as *Helicobacter pylori* have been detected in the placentas of women with preeclampsia (Ponzetto et al., 2006). In a study comparing the placentas of 55 women with preeclampsia to 55 control group placentas, seven PCR-positive samples were found in the preeclampsia group, which included bacteria associated with gastrointestinal infections such as *Bacillus cereus*, *Listeria*, *Salmonella*, *Escherichia*, those associated with respiratory infections like *Klebsiella pneumonia* and *Anoxybacillus*, and those related to periodontitis such as *Variovorax*, *Prevotella*, *Porphyromonas*, and *Dialister* (Amarasekara et al., 2015). Changes in the oral microbiota have been associated with the development of preeclampsia. Pathogenic bacteria in the oral cavity may enter the placenta through the bloodstream or other pathways, triggering an inflammatory response and exacerbating the condition of preeclampsia. Placental samples collected from 16 preeclamptic women and 14 healthy pregnant women undergoing cesarean section revealed bacteria similar to periodontal pathogens, including *Actinobacillus actinomycetemcomitans*, *Fusobacterium nucleatum ssp*., *Porphyromonas gingivalis*, *Prevotella intermedia*, *Tannerella forsythensis*, and *Treponema denticola*. Quantitative measurements of these bacteria showed higher bacterial counts in the preeclampsia group (Barak et al., 2007). It has been reported that in placentas of preeclamptic women with periodontitis, there is an association between *P. gingivalis* and *P. intermedia* with increased expression of TLR-4 and NF-κB (Parthiban et al., 2018).

The placenta, as an organ directly affecting the maternal-fetal interface, likely harbors unique microbial communities and their derivatives that can trigger inflammation. Microorganisms in the placenta can promote the release of anti-angiogenic factors like soluble fms-like tyrosine kinase-1 (sFlt-1) while reducing the release of pro-angiogenic factors such as placental growth factor (PlGF) and vascular endothelial growth factor (VEGF), affecting the vascularization status and causing endothelial dysfunction, ultimately leading to the clinical manifestations of Preeclampsia (Villar et al., 2006; Olaniyi et al., 2020). Nizyaeva et al. analyzed 20 cases of preeclampsia in pregnant women and 12 cases of normal pregnancy. The results showed that 45% of the Preeclampsia group exhibited chronic villitis, compared to only 8% in the control group. After culturing all placentas, pathological microbial growth (including non-group *B streptococci* or group *B streptococci* and *Staphylococcus epidermidis*) was found in 25% of placentas in the preeclampsia group, while all placentas in the control group showed negative culture results (Nizyaeva et al., 2017). Therefore, microorganisms may participate in inflammatory responses, supporting the role of microbial interactions in the pathogenesis of preeclampsia.

Metabolites such as lipopolysaccharides from Gram-negative bacteria and lipoteichoic acid or peptidoglycan from Gram-positive bacteria can activate Toll-like receptors (TLRs), typically TLR-2 and TLR-4. This activation leads to the activation of NF-kB through a series of intermediate steps, ultimately inducing inflammation, which collectively contributes to the onset of preeclampsia (Lin et al., 2012; Kell and Kenny, 2016). Changes in the composition of the placental microbiota or alterations in the dominance of certain bacteria in preeclampsia can disrupt this relatively balanced microenvironment. Additionally, dysbiosis in the placental microbiota may disrupt the metabolism of tryptophan and fatty acids, exacerbating inflammatory stimuli and thereby exacerbating the preeclampsia disease process (Olaniyi et al., 2020). In summary, alterations in the placental microbiota may be a potential factor contributing to the development of preeclampsia. Further research is needed to deepen our understanding of the relationship between placental microbiota and preeclampsia, providing new insights and strategies for the prevention and treatment of preeclampsia.

### 5.3 Placental microbiota and fetal growth restriction

FGR is a serious complication of pregnancy, broadly defined as impaired fetal growth potential, with estimated fetal weight below the 10th percentile for gestational age fetuses. The etiology and pathogenesis of intrauterine growth restriction (IUGR) remain incompletely understood, despite its significant impact on perinatal morbidity and mortality (Romo et al., 2009; Nardozza et al., 2017). Recent research has begun to explore the potential role of placental microbiota in FGR. Stupak et al. (Stupak and Kwaśniewski, 2023) utilized proteomics and bioinformatics analysis to investigate the placental biomass from 18 physiological pregnancies and 18 pregnancies complicated by FGR. Their findings revealed distinct differences in the microbial composition between the two groups. In the FGR group, there was an increased presence of *Actinopolyspora erythraea*, *Listeria costaricensis*, *Escherichia coli*, *Methylobacterium*, *Acidobacteria bacterium*, *Bacteroidetes bacterium*, *Paenisporsarcina* sp., *Thiodiazotropha endoloripes*, and *Clostridiales bacterium*. In contrast, the normal pregnancy group exhibited higher levels of *Flavobacterial bacterium*, *Aureimonas sp*., and *Bacillus cereus* (Stupak and Kwaśniewski, 2023). These distinct placental microbiota characteristics in FGR pregnancies suggest a potential link between microbial dysbiosis and impaired fetal growth.

There is emerging evidence that links the placental microbiome to birth weight in full-term newborns, with variations in microbial composition potentially influencing fetal growth outcomes. Specifically, a study has identified significant differences in the placental microbiota at both the phylum and genus levels between infants with low birth weight and those with normal birth weight (Zheng et al., 2015). A reduction in the overall richness of the placental microbiota has been associated with lower birth weights, suggesting that a less diverse microbiome may contribute to suboptimal fetal growth. Notably, the abundance of Lactobacilli in the placental microbiota shows a positive correlation with birth weight, implying that these beneficial bacteria may support healthier fetal development (Zheng et al., 2015). *Lactobacilli* are well-known for their probiotic properties, including their capacity to modulate inflammation and maintain microbial balance. This raises the possibility that supplementing with *Lactobacilli* could be a potential therapeutic strategy for optimizing placental health and, consequently, fetal growth by enhancing the placental microbiota (Naito et al., 2011; Ejtahed et al., 2012). Further research is needed to confirm these findings and explore the potential for targeted probiotic interventions during pregnancy.

### 5.4 Placental microbiota and gestational diabetes mellitus

The placenta plays a crucial role as the connection between the mother and the fetus, ensuring proper nutrient exchange, hormonal signaling, and immune protection. In pregnancies affected by GDM, the placenta often exhibits distinct morphological changes, including increased size, immature villi, and various vascular abnormalities compared to normal pregnancies. These alterations may significantly impact the placental microbiota, posing short-term and long-term health risks to both the mother and the fetus (Johns et al., 2018; Ehlers et al., 2021; Sara et al., 2022; Xie et al., 2022). However, the precise mechanisms through which a mother’s GDM status influences the placental microbiota are not yet fully understood. One hypothesis suggests that the hyperglycemic state characteristic of GDM induces a pro-inflammatory and oxidative stress environment, which may affect the placental microbiota. This state of chronic inflammation and oxidative stress can disrupt the delicate balance of the placental microbiota, leading to dysbiosis. Another possible mechanism is the oral-placental route, where oral pathogens or their metabolites might translocate to the placenta, thereby altering its microbial composition (Sara et al., 2022).

Research indicates that the placental microbiota in women with GDM differs from that in women with normal blood sugar levels. For example, women with GDM tend to exhibit a reduction in the abundance of the order *Pseudomonadales* and the genus *Acinetobacter* (Bassols et al., 2016). The decrease in *Acinetobacter* abundance has been associated with lower blood eosinophil counts and reduced expression of several anti-inflammatory genes in the placenta, including interleukin-10. *Acinetobacter* is believed to play a role in regulating the maternal immune system, contributing to an anti-inflammatory environment in the placenta, which is crucial for maintaining a healthy pregnancy. Some studies have also suggested that the placental microbiota in GDM may exhibit greater diversity compared to pregnancies with normal blood sugar levels (Tang et al., 2020). This increased microbial diversity could reflect an altered immunologic tolerance in the placenta, possibly driven by the hyperglycemic environment. The dysbiosis observed in GDM may influence the maternal-fetal immune interaction, contributing to the pathophysiology of GDM (Zheng et al., 2017). The placental microbiota in both GDM and control groups primarily consists of four phyla: *Bacteroidetes*, *Firmicutes*, *Actinobacteria*, and *Proteobacteria*. However, specific differences in genus abundance have been observed between the placentas of women with GDM and those with normal blood sugar levels, including higher levels of *Ruminococcus*, *Coprococcus*, *Paraprevotella*, and *Lactobacillus*, and lower levels of *Veillonella* in GDM (Tang et al., 2020). These microbial shifts may be linked to the inflammatory state induced by GDM, as well as alterations in placental immune signaling pathways.

Variations in placental microbiota profiles may be associated with clinical characteristics in both the mother and the infant, such as umbilical cord insulin, IGF-1, and leptin levels (Zheng et al., 2017). These associations suggest that the placental microbiota could play a role in regulating fetal glucose metabolism, development, and growth, potentially influencing outcomes related to fetal and neonatal health. The altered microbiota in GDM pregnancies might contribute to dysregulated fetal metabolic programming, predisposing the offspring to metabolic disorders later in life.

## 6 Interventions to modulate the placental microbiota community

Although there is a growing body of evidence supporting the role of probiotics in promoting gut microbiota balance, direct research on their impact on the placental microbiota remains somewhat limited (Sanders, 2008; Chen et al., 2019; Borka Balas et al., 2023). While some studies have started to investigate the potential effects of probiotics on maternal health and infant development, the specific relationship between probiotics and the placental microbiota is not yet well understood. For instance, there is evidence suggesting that probiotic intake during pregnancy may be associated with a reduced risk of gestational diabetes mellitus (GDM) and other pregnancy-related complications, as well as influencing the development of the infant’s immune system (Luoto et al., 2010; Brantsaeter et al., 2011; Asemi et al., 2013; Kwok et al., 2022; Alsharairi and Li, 2023; Tian et al., 2023). In one study, genetically labeled *Enterococcus faecium* was orally administered to pregnant mice, and traces of the bacteria were detected in the meconium of pups delivered by cesarean section (Jiménez et al., 2008). This finding suggests a potential mechanism of translocation where probiotic bacteria or their metabolic products might traverse the maternal gut, possibly *via* the bloodstream, to reach the fetal environment.

Furthermore, probiotic strains from *Lactobacillus* and *Bifidobacterium* have been identified in human placentas, and maternal supplementation with probiotics has been shown to modulate the expression of TLR-related genes in both the placenta and fetal intestines (Rautava et al., 2012a). This modulation of TLR-related genes indicates that probiotics may influence immune signaling pathways within the placenta, potentially altering the maternal-fetal immune dialogue and impacting fetal development. Probiotics may also contribute to the maintenance of a balanced microbial community in the maternal reproductive tract, which can indirectly affect the placenta by modulating the microbial environment during pregnancy (Liang et al., 2021).

These interactions suggest a possible mechanism through which probiotics could influence placental health by maintaining or restoring microbial balance, reducing inflammation, and modulating immune responses. While these studies provide some indirect evidence for the role of probiotics in maternal health and fetal development, research directly addressing the impact of probiotics on the placental microbiota is still relatively limited. Therefore, further studies are needed to elucidate the precise mechanisms by which probiotics might influence the placental microbiota and their implications for maternal and fetal health.

## 7 Conclusion and perspectives

Human health is influenced by two fundamental factors: the human genome and the human microbiome. The insights gained from the Human Microbiome Project offer valuable opportunities to identify molecular markers that could enhance early disease diagnosis and treatment, ultimately paving the way for personalized precision medicine. By incorporating these findings, medicine can become more tailored, providing patients with treatments that are both accurate and effective.

The placenta, essential for fetal development, has long been underappreciated despite its significant impact on the health of both the mother and baby. Recent studies have proposed the presence of distinct microbial communities within the placenta, which may play vital roles in maternal and fetal health. However, the concept of a placental microbiome is still a subject of debate, with some researchers questioning whether the microbial signatures detected in placental tissue truly represent an active microbiota or are instead the result of contamination or translocation from other body sites. If the placenta harbors microbial communities, understanding their composition, origin, and potential influence could be essential for preventing adverse pregnancy outcomes, supporting normal newborn development, and promoting long-term health for the offspring. Research into the changes and dynamics of placental microbiota throughout pregnancy is essential for comprehending their formation and evolution. While our understanding of the diversity, abundance, and metabolic functions of placental microbiota is still evolving, further studies are necessary to explore the intricate relationship between these microbial communities and local immune responses within the placenta. Investigating how these microbes interact with the placental immune system, including their influence on immune cell differentiation, activity, and function, as well as how immune cells regulate microbial growth and metabolism, is an area ripe for exploration. Using animal models or *in vitro* systems could provide deeper insights into these mechanisms and validate emerging hypotheses.

Our study highlights the impact of delivery methods on the placental microbiome. One potential limitation of the studies analyzed is the influence of the mode of delivery on placental microbiome contamination. Vaginal deliveries, in particular, are associated with a higher likelihood of microbial contamination from the birth canal, which could alter the microbiome profile of the placenta. This factor should be carefully considered when interpreting the results of placental microbiome analyses, and future studies may benefit from further exploration of delivery mode as a confounding variable. Additionally, there are several other limitations to consider. First, the variability in sample sizes across studies may introduce biases, affecting the robustness and generalizability of the findings. Second, discrepancies in methodologies, including the use of different sequencing platforms and bioinformatics pipelines, could contribute to inconsistencies in microbial community profiling. Lastly, while our analysis focused primarily on the microbiota’s composition, we were unable to fully investigate the functional implications of these microbial communities on maternal and neonatal health. To address these gaps, future studies should aim to standardize methodological approaches and explore the functional roles of the placental microbiome, particularly in relation to different delivery methods.

Emerging evidence suggests that abnormalities in placental microbiota might be linked to certain pregnancy complications and fetal developmental issues, such as preterm birth, gestational hypertension, and intrauterine growth restriction. Further research could delve into the potential connections between these microbial imbalances and related health outcomes, identifying specific differences in placental microbiota that may be associated with complications and uncovering potential pathogenic bacteria. By addressing maternal microbiota dysbiosis before birth, it might be possible to enhance the effectiveness of treatments for adverse pregnancy outcomes. Interventions that focus on modulating the composition or function of placental microbiota to support immune balance could play a role in the prevention or management of these conditions. For instance, probiotics might be considered as a strategy to support pregnancy health in the context of abnormal placental microbiota. Additionally, by addressing oral microbiota, which shares similarities with placental microbiota, and mitigating issues like periodontal disease, it may be possible to reduce the risk of pregnancy complications through the disruption of microbial transmission pathways, such as the “oral-placental axis.”
